# Mediated autobiographical remembering in the digital age: insights from an experimental think-aloud study

**DOI:** 10.1186/s41235-025-00627-4

**Published:** 2025-05-01

**Authors:** Fabian Hutmacher, Beate Conrad, Markus Appel, Stephan Schwan

**Affiliations:** 1 Human-Computer-Media Institute, Julius-Maximilians-Universität Würzburg, Oswald-Külpe-Weg 82, 97074 Würzburg, Germany; 2 Leibniz-Institut Für Wissensmedien, Tübingen, Germany

**Keywords:** Autobiographical memory, Digital age, AMEDIA-model, Think-aloud method

## Abstract

Autobiographical remembering may undergo significant transformations in the digital age, in which the omnipresence of digital tools has led to an increased density of recorded life episodes. To gain deeper insights into these processes, we conducted an experimental think-aloud study in which participants (*N* = 41) had to remember an important day and a random day that happened about one year ago. As the results demonstrate, participants repeatedly switched between information stored in their minds and information stored in external resources when remembering these events, with digital resources playing a particularly prominent role. The number of changes between internal memories and external resources as well as the number of digital resources that individuals used were higher when remembering the random day. In sum, this suggests that the iterative combination of information stored in one’s mind and information stored in external resources can be considered a potentially symbiotic process.

**Supplementary Information:**

The online version contains supplementary material available at 10.1186/s41235-025-00627-4.

## Significance statement

Never before have humans preserved more information about their lives than today. In other words, the constant accumulation of data from different media sources has led to an increased density of recorded life episodes. It seems intuitively plausible to assume that this profoundly shapes the way we remember and reconstruct our lives. How this impact may look like is largely unchartered territory so far. To integrate the available evidence and to provide a framework for future research, we have recently proposed a model for autobiographical remembering in the digital age. In this model, we assume that autobiographical remembering is an iterative process that is based on the combination of information stored in one’s mind and information stored in external resources. In addition, we also assume that digital external resources play a key role for autobiographical remembering in our modern-day societies. The present study tested and confirmed these assumptions. Apart from that, we also varied whether participants had to remember and reconstruct an important day or a random day. Participants used more external resources and changed more frequently between information stored in their minds and information stored in external resources when remembering the random day. In sum, the present study provides compelling evidence for the importance of considering the interplay between information stored in our minds and information stored in external resources as well as the role of digital resources when it comes to autobiographical remembering in the twenty-first century.

## Introduction

Never before have humans preserved more information about their lives than today in our digital societies—just think about the pictures that we take using our smartphones, the e-mails and *WhatsApp*-messages that we write, the information that we post on social media (e.g., *BeReal*, *Instagram*, *Snapchat*), or the data that we record using health trackers and wearables (see, e.g., Cho et al., 2023). In other words, the constant accumulation of data from different media sources has led to an increased density of recorded life episodes (Heersmink & Carter, 2020; Kalnikaitė & Whittaker, 2012). It seems intuitively plausible to assume that this profoundly shapes the way we remember and reconstruct our lives. To date, however, research on autobiographical remembering in the digital age is still nascent.

Building on previous discussions about the extended mind and technology-mediated memory (e.g., Clark & Chalmers, 1998; Eliseev & Marsh, 2021; Finley & Naaz, 2023; Finley et al., 2018; Heersmink, 2022; Marsh & Rajaram, 2019; Schönpflug & Esser, 1995; Sutton, 2010), transactive memory (Wegner et al., 1985; for a review, see, e.g., Huebner, 2016; Peltokorpi, 2008), and electronic memory (Clowes, 2013; see also Bell & Gemmell, 2009), we recently suggested a model for Autobiographical Memory in the Digital Age (AMEDIA-Model; Hutmacher et al., 2024a, 2024b) that tries to integrate the available evidence and to provide a framework for future research. In short, the AMEDIA-Model views autobiographical remembering as the process of combining information stored in one’s mind (e.g., what one remembers about one’s last birthday) and information stored in external resources (e.g., photos, videos, instant messages, and diary entries about one’s last birthday). Crucially, the interplay between information stored in one’s mind and external resources is dynamic and iterative rather than static and unidirectional: For instance, you could pick up your phone to scroll through the photo gallery because you are not sure anymore whether a certain friend attended the birthday party. Assume that the photographs confirm that the friend had indeed shown up. While scrolling through the photographs, however, you also stumble upon a picture depicting the different foods and snacks that you had prepared. Seeing this, you remember that another friend loved the salad dressing and asked for the recipe—and you also remember that this friend has been going through difficult times lately, and so on. Put in one sentence, the AMEDIA-Model hypothesizes that autobiographical remembering (in the digital age) is an iterative process that is based on the combination of information stored in one’s mind and information stored in external resources. However, this fundamental assumption underlying the AMEDIA-Model has not been tested empirically so far.

The same applies to a second assumption, namely the importance of digital external resources for autobiographical remembering in our modern-day societies: That individuals use external resources for (autobiographical) remembering is not a peculiarity of the digital age but something that humans have been doing for thousands of years, relying on various techniques from cave paintings and oral traditions to archives, monuments, and printed books to paper diaries and photo albums (e.g., Donald, 1991, 2001; Heersmink, in press). In addition to such *non-digital resources*, people routinely also refer to other individuals as *social resources* for remembering (e.g., “You were at my birthday party. Do you remember whether …?”). However, the new *digital resources* that have become available over the last years and decades differ significantly from these other resources—not only because they have overcome previous storage limits but also because they provide a combination of different kinds of media sources (e.g., photos, videos, audio recordings, text, quantitative parameters) and because they lead to a searchable database that can often be accessed anywhere at any time (Hutmacher et al., 2024a; for the distinction between the different kinds of resources, see also Finley et al., 2018). Against this background and given the worldwide proliferation of digital technologies, it seems plausible to hypothesize that digital external resources play an important role when it comes to remembering autobiographical events in the digital age.

Apart from these general questions about the interplay of internal memories and information stored in external resources as well as the importance of digital resources, it seems plausible to assume that these processes are shaped by the kinds of events that individuals try to remember. Given that individuals usually have more detailed and more vivid internal memories about important autobiographical events (e.g., their last birthday) compared to less important autobiographical events (e.g., autobiographical memories regarding a random day one year ago), one could speculate, for instance, that this also impacts the way individuals interact with external resources when it comes to remembering these events. On the one hand, one might think that individuals will have more external information available for important events compared to less important events; on the other hand, one could also think that individuals will rely more on external resources for relatively unimportant events compared to important events due to a lack of retrievable internal memories.

In order to test the predictions derived from the AMEDIA-Model and to investigate that the kind of event plays in this context, we conducted an experimental think-aloud study in which participants were asked to remember and reconstruct an important day and a random day while being in their usual home environment in which they had access to all external resources they would usually have access to. That is, following calls to embrace methodological pluralism in general (Araujo & Osbeck, 2023; Hutmacher, 2023; Hutmacher & Franz, 2025; Mayrhofer & Hutmacher, 2020; Zitzmann & Loreth, 2021) and to move beyond purely laboratory-based paradigms in research on autobiographical memory in particular (Brockmeier, 2015; Fivush, 2013; Hutmacher et al., 2024a; Yamashiro & Roediger, 2019; see also Grysman, 2024) as well as following exemplary studies in this direction that have been published in recent years (e.g., Armstrong et al., 2023; Barnwell et al., 2023; Hutmacher et al., 2023; Johnson & Morley, 2021; Jungselius & Weilenmann, 2023; Martin et al., 2022; Talaifar, 2024), we decided to create a setting that closely resembles real-life situations that individuals may encounter when thinking about events from their past (e.g., “How exactly did I celebrate my last birthday?”, “What did I do Friday afternoon two weeks ago?”). In addition, relying on the think-aloud method allowed us to obtain verbal reports that closely mirror individuals’ actual thought processes and behaviors and that go far beyond the information that can be collected through self-reports alone (cf. Baumeister et al., 2007). Taken together, the present study allowed us to gain important insights into the way external resources shape autobiographical remembering in the twenty-first century.

## Method

### Participants

As this was the first study investigating autobiographical remembering in the digital age using a think-aloud protocol, there were no effect sizes available from previous studies on which we could have based our power analysis. As we were particularly interested in comparing our results for two kinds of events (important day vs. random day), we decided that we wanted to have enough power (0.80) to detect a medium-sized effect (*d* = 0.50) in a *t*-test with dependent samples (*α* = 0.05, two-tailed). Using G*Power 3.1.9.7 (Faul et al., 2007), we calculated a minimum sample size of 34 participants. To account for potential exclusions, we decided to collect data from at least 40 participants. Participants were recruited through the participant recruitment system used by the University of Würzburg (Germany). All participants received course credit. In total, 41 participants completed the study (18–33 years, *M* = 22.73, *SD* = 3.26, 10 male, 30 female, 1 diverse). In terms of educational attainment, one participant had completed secondary education, 37 participants had completed high-school, and three participants had a bachelor’s degree. The study was conducted between March 22 and May 23, 2024. This study was not preregistered.

### Design and procedure

The experiment was conducted in two sessions per participant that were scheduled one week apart. In both sessions, a videoconferencing tool was used. Participants were told beforehand that they should be in their usual home environment for both sessions. During one of the sessions, participants were asked to remember and reconstruct an *important day* in as much detail as possible; during the other session, participants were asked to remember and reconstruct a *random day* in as much detail as possible. During which session participants had to remember the important day and the random day, respectively, was counterbalanced across participants. As far as the important day is concerned, participants were asked to remember their last birthday (if their last birthday occurred more than six months before the session) or their second last birthday (if their last birthday occurred less than six months before the session). This way, participants on average remembered an important day from about one year ago. We chose the birthday as an important day as we assumed that most participants would find their birthday important and consider it a day that stands out from the rest of the year. As far as the random day is concerned, participants were asked to remember the day exactly one year before the session. That is, if the session with the random day took place on May 14, 2024, the participant would be asked to remember May 14, 2023.

In the beginning of the first session, participants provided informed consent and answered demographic questions using an online questionnaire. Both sessions were conducted using the think-aloud method. In setting up the instructions, we followed extant recommendations (Ericsson & Simon, 1993; see also Eccles & Arsal, 2017; Noushad et al., 2024). That is, participants were instructed to verbalize everything they think from the point when they receive the instructions to the point when they complete the task. Participants were told that they should not think about how to express their thoughts and that there was no need to explain their thoughts to the interviewer. Instead, participants were told to behave as if they were alone in a room, talking to themselves. Participants were also told that the interviewer would remain passive while the participants perform the think-aloud task, with the only exception that the interviewer would remind them to think aloud in case they should forget the instruction. To familiarize participants with the think-aloud method, two practice tasks were performed: While the first practice task was completely unrelated to autobiographical remembering (“Please calculate: What is 24 × 36?”), the second practice task was related to autobiographical remembering but unrelated to remembering the important day and the random day (“When and with whom did you eat pizza for the last time?”). These practice tasks were only performed during the first session as we assumed that participants would be sufficiently familiar with the procedure during the second session. In the second session, the interviewer repeated the basic instructions regarding the think-aloud procedure.

Next, participants were informed that they will be asked to remember and reconstruct a specific day in as much detail as possible without already revealing which day it will be. Participants were told that they would have up to 15 min to perform this task. It was emphasized that this time frame was not meant to create time pressure but to restrict the session to a reasonable length. Participants were told that they can finish the task earlier if they have the impression that there is nothing more to be remembered. In addition, participants were told that the interviewer would inform them when the time is up so that they do not have to pay attention to the 15-min time frame. Participants were told that they are allowed to use all kinds of digital and non-digital resources that they may want to use to complete the task. It was emphasized that participants are allowed to stand up and even to leave the room in case this should be necessary to get access to a certain resource or to talk to another person. With respect to the day to be remembered, it was emphasized that a reconstruction of this day could—among others—give answers to the following questions: Where were you? What happened? With whom did you spend the day? How did you feel during the day? Which thoughts and feelings did you experience during the day? In case participants encountered information that they found too personal to share, they were asked to give a factually correct but more abstract description that left out the details that they did not want to be recorded. Before being informed which specific day to remember, participants were given the opportunity to ask questions about the procedure. After completing the think-aloud task, the interviewer was allowed to ask ad-hoc questions to ensure that all changes between different external resources and the source from which participants retrieved a certain piece of information can be identified correctly when looking at the interview transcripts later.

### Data analysis

The interviews were recorded and transcribed verbatim. We processed and analyzed the transcripts with MAXQDA 2022 (VERBI software). First, a codebook was created: The overarching categories for analysis (i.e., changes between internal memory and external resources, kinds of external resources, retrieved information; for details, see below) were already determined prior to data collection. The sub-categories within these overarching categories (e.g., which digital resources participants used) were created based on initiating text work (e.g., Kuckartz, 2019); that is, an initial read-through of the material was used as an inductive coding strategy to deduce the different sub-categories. More specifically, two coders first familiarized themselves with the data independently from one another. Then, each coder created an initial draft for a codebook. These two drafts were discussed between the two coders and integrated into a final codebook. The final codebook included definitions of each category as well as examples and explicit coding rules.

Next, the data were coded based on the codebook. To ensure the objectivity of the coding process, about 20% of the data (i.e., both sessions from eight participants) were coded by the two coders independently from one another. After completing the independent coding, an interrater reliability was calculated (Brennan & Prediger, 1981). Two coded segments of the material were considered to match when the overlapping rate of the two codes was 90% or higher. The interrater reliability (including the additional analyses reported below) indicated an almost perfect agreement, *κ* = 0.86 (cf. Landis & Koch, 1977). The coders discussed the differences with respect to independently coded interviews and agreed upon a final solution. Given the substantial agreement between the two coders, one coder coded the remaining material. However, open questions and potentially ambiguous sections were discussed between the two coders. After completing the coding procedure, code frequencies were calculated to enable quantitative analysis. In the following, we describe the different variables that resulted from the qualitative data analysis and that were then used for the statistical analyses.

#### Changes between internal processes and external resources

##### Number of changes and time to first change

It was coded how often participants changed between internal processes and external resources. Three kinds of changes were distinguished: *changes from internal processes to an external resource*, that is, situations in which an internal thought process or memory leads to the use of an external resources (e.g., “I guess I had a doctor’s appointment in the afternoon, but I will check that in my calendar”); *changes from an external resource to internal processes*, that is, situations in which information encountered in an external resource leads to an internal thought process or memory (e.g., “Now that I see this picture from the restaurant, I also remember that we went to a bar around the corner later”); and *changes from one external resource to another external resource*, that is, situations in which two external resources are used one after another without detailed thought processes or memories being described in-between (e.g., “Ok, there is nothing about this afternoon in my calendar, so I will check my mails next”). The frequencies for these three kinds of changes were summed up to get an estimate for the overall number of changes between internal processes and external resources. During our analysis, we assumed—for logical reasons—that the different kinds of changes form an unbroken chain in which a change from an internal process to an external resource is always followed by a change from an external resource to an internal process (or a change to another external resource) and vice versa. In addition, we also assumed that the first change during the think-aloud procedure is always a change from internal processes to external resources (i.e., the moment in which participants decided to use an external resource for the first time) and that the last change during the think-aloud procedure is always a change from external resources to internal processes (i.e., the moment in which participant decided to end the task). Ultimately, this implies that the total number of changes from an internal process to an external resource is the same as the total number of changes from an external resource to an internal process. Apart from documenting the different kinds of changes, we also determined how long it took participants from receiving the instructions to consulting an external resource for the first time.

##### Additional analyses

To gain deeper insights into the processes underlying the different kinds of changes, we conducted additional analyses.^1^ As far as the changes from internal processes to an external resource are concerned, we distinguished three cases (based on our data): (a) the change to an external resource is initiated because participants have *no internal memory* and hope to find information in an external resource (e.g., “I really do not remember, what I did on this day; let me check my phone”); (b) the change to an external resource is initiated because participants have a *vague internal memory*, but are lacking details, which they hope to find in an external resource (e.g., “I think I spent the day with my usual group of friends, but I am not exactly sure what we did”); (c) the change to an external resource is initiated because participants have a *detailed internal memory* but want to confirm this internal memory (e.g., “I am pretty sure that I went to a restaurant with my usual group of friends that day, but let me check whether this is actually true”). As far as the changes from an external resource to internal memory are concerned, we first distinguished whether consulting an external resource was *successful* (i.e., participants found useful information in the external resource) or *unsuccessful* (i.e., participants did not find useful information in the external resource). In case consulting an external resource was successful, we further distinguished whether the external resource (a) merely *confirmed previous knowledge* (e.g., “Yes, we went to a restaurant that day, exactly as I had thought”) or whether it (b) led to *new insights* and/or triggered new internal memories (e.g., “Oh, and I had this fancy cocktail, I had completely forgotten that – now I remember!”).

#### Kinds of external resources

It was coded, which *social resources* (e.g., family, partner), *non-digital resources* (e.g., paper diary, paper calendar), and *digital resources* (e.g., smartphone photo gallery, digital calendar, instant messaging apps, social media) individuals used. Based on this, we calculated the number of social, non-digital, and digital resources as well as the total number of different external resources that individuals used.

#### Retrieved information

It was coded whether the information that individuals retrieved was based on a mental representation (*internal retrieval*) or whether it was solely based on information stored in an external resource (*external retrieval*). Note that internal retrieval comprises cases of unaided internal retrieval (i.e., internal retrieval of information without the use of an external resource; e.g., “I remember spending that evening in a restaurant”) and aided internal retrieval (i.e., internal retrieval of information through the use of an external resource; e.g., “Now that I see it on the picture, I also remember spending that evening in a restaurant”). As it was not always clear from the think-aloud protocols whether participants remembered a certain piece of information on their own or whether their recorded data helped them to remember, we decided to use *internal retrieval* as an umbrella category.

To capture the full range of different kinds of information that individuals remember, additional sub-categories were used to code whether the retrieved information concerned *external characteristics* (i.e., information about the *place* where an event happened, information about the details of an *event*, information about the *persons* who were present, and information about the *conversational content*) or *internal states* (i.e., information about *thoughts* and *feelings*). For instance, the sentence “On this picture, I can see that I spent a nice evening with friends in restaurant X in city Y” would be coded in the category *external retrieval* (“on this picture”) and, more specifically, in the sub-categories *place* (“in restaurant X in city Y”), *event* (“a nice evening with friends in restaurant X”), and *persons* (“with friends”). If the sentence had begun with “I remember that” instead of “On this picture”, the same sub-categories would have been assigned, but under the higher-order category *internal retrieval*. Note that comparing the number of external characteristics and the number of internal states that participants remembered is difficult because the number of sub-categories was different (four for external characteristics and two for internal states) and because these sub-categories represent phenomenologically diverse kinds of information (e.g., remembering a certain feeling vs. remembering the name of a restaurant). Against this background, we decided to sum up the number of external characteristics and the number of internal states that participants remembered to get a joint estimate for information remembered through internal retrieval and external retrieval, respectively. However, we report the frequencies of the different types of information (i.e., the different kinds of internal states and external characteristics) for the different types of retrieval (i.e., internal versus external retrieval) and the different types of events (i.e., random day vs. important day) in Table 4. Note also that each piece of information was only counted once, even if it was mentioned several times during the think-aloud procedure (e.g., when the same restaurant was mentioned three times, this was only counted as one piece of information) so that the retrieved amount of information equals the number of different pieces of information that participants retrieved. In addition to counting the amount of information that participants retrieved, we determined the total amount of time that participants spent performing the think-aloud procedure.

## Results

All statistical analyses were performed using JASP (2024) (Version 0.19.3).

### Time spent on the think-aloud procedure

The time that participants spent on performing the think-aloud procedure did not differ significantly between remembering the important day (*M* = 6.87 min, *SD* = 3.11) and remembering the random day (*M* = 7.47 min, *SD* = 3.45), *t*(40) = 1.31, *p* = 0.098, *d* = 0.21, 95% CI [*t*0.11, 0.51]. As there was a descriptive tendency that participants spent more time on remembering the random day, we nevertheless checked whether correcting the parameters analyzed in the following for the time spent on the task changes the results in any meaningful way. This was not the case: All significances remained the same. Hence, these analyses are reported in the Supplemental Material.

### Changes between internal processes and external resources

#### Number of changes and time to first change

Participants repeatedly changed between internal processes and external resources, both when remembering the important day (*M* = 5.90, *SD* = 4.80) and when remembering the random day (*M* = 8.05, *SD* = 5.27). Overall, and as indicated by a *t*-test for dependent samples, participants changed more frequently between internal processes and external resources when remembering the random day compared to remembering the important day, *t*(40) = 3.82, *p* < 0.001, *d* = 0.60, 95% CI [0.26, 0.93]. As indicated by another *t*-test for dependent samples, it took participants less time until they consulted an external resource for the first time when remembering the random day (*M* = 0.23 min, *SD* = 0.25) compared to remembering the important day (*M* = 1.21 min, *SD* = 1.54), *t*(36) = 3.86, *p* < 0.001, *d* = 0.64, 95% CI [0.28, 0.99].^2^ In sum, this suggests that participants were more reliant on external resources when remembering the random day.

#### Additional analyses

An overview of the number and kind of changes between internal processes and external resources is provided in Table 1. First, we ran a 2 × 3  repeated-measures ANOVA to determine whether the *number of changes from an internal process to an external resource* changed depending on the *kind of event* (important day vs. random day) and the *reason for the change* (no internal memory vs. vague internal memory vs. detailed internal memory). We found a significant main effect for the kind of event, *F*(1, 40) = 7.84, *p* = 0.008, $\omega_{p}^{2} = 0.0{25}$, a significant main effect for the reason of the change, *F*(2, 80) = 38.49, *p* < 0.001, $\omega_{p}^{2} = 0.326$, and a significant interaction between the two factors, *F*(2, 80) = 17.80, *p* < 0.001, $\omega_{p}^{2} = 0.159$.^3^

**Table 1:** Number and kind of changes between internal processes and external resources

	Important day		Random day	
	M	SD	M	SD
*Changes from internal to external*	2.71	2.11	3.44	2.16
No internal memory	0.46	0.67	1.83	1.53
Vague internal memory	2.00	1.69	1.56	1.47
Detailed internal memory	0.24	0.54	0.05	0.22
*Changes from external to internal*	2.71	2.11	3.44	2.16
Successful: confirming	0.44	0.59	0.15	0.53
Successful: new	1.68	1.69	2.00	1.24
Unsuccessful	0.59	0.89	1.29	1.44
*Changes from external to external*	0.49	0.90	1.17	1.53
*Total number of changes*	5.90	4.80	8.05	5.27

To better understand this interaction, we additionally calculated post-hoc *t*-tests using a Bonferroni correction. As far as the important day is concerned, more changes from an internal process to an external resource were initiated based on a vague internal memory than based on no internal memory, *t*(40) = 6.22, *p* < 0.001, *d* = 1.32, 95% CI [0.51, 2.13], or a detailed internal memory,* t*(40) = 6.56, *p* < 0.001, *d* = 1.51, 95% CI [0.62, 2.40]. There was no significant difference between the amount of changes from an internal process to an external resource initiated based on no internal memory and a detailed internal memory, *t*(40) = 1.50, *p* = 0.423, *d* = 0.19, 95% CI [*t*0.21, 0.59]. That is, for the important day, the dominant reason for consulting an external resource was having a vague internal memory. As far as the random day is concerned, more changes from an internal process to an external resource were initiated based on no internal memory than based on a detailed internal memory, *t*(40) = 7.25, *p* < 0.001, *d* = 1.53, 95% CI [0.68, 2.38], as well as based on a vague internal memory than based on a detailed internal memory,* t*(40) = 6.67, *p* < 0.001, *d* = 1.30, 95% CI [0.54, 2.06]. There was no significant difference between the amount of changes from an internal process to an external resource initiated based on no internal memory and a vague internal memory, *t*(40) = 0.82, *p* = 1.00, *d* = 0.23, 95% CI [− 0.65, 1.11]. That is, for the random day, the dominant reasons for consulting an external resource were having either no internal memory or a vague internal memory. In addition, there were more changes from an internal process to an external resource based on no internal memory for the random day compared to the important day, *t*(40) = 6.63, *p* < 0.001, *d* = 1.17, 95% CI [0.49, 1.86], more changes based on a detailed internal memory for the important day compared to the random day, *t*(40) = 2.24, *p* = 0.031, *d* = 0.17, 95% CI [− 0.07, 0.41], and no significant difference between the number of changes based on a vague internal memory for the important day compared to the random day, *t*(40) = 1.55, *p* = 0.130, *d* = 0.38, 95% CI [− 0.40, 1.15].

Next, we ran another 2 × 3  repeated-measures ANOVA to determine whether the *number of changes from an external resource to an internal resource* varied as a function of the *kind of event* (important day vs. random day) and the *reason for the end of the consultation of the external resource* (confirming previous knowledge vs. new insights vs. unsuccessful consultation). We found a significant main effect for the kind of event, *F*(1, 40) = 7.84, *p* = 0.008, $\omega_{p}^{2} = 0.0{25}$, a significant main effect for the reason of the change, *F*(2, 80) = 35.52, *p* < 0.001, $\omega_{p}^{2} = 0.305$, and a significant interaction between the two factors, *F*(2, 80) = 5.00, *p* < 0.009, $\omega_{p}^{2} = 0.0{42}$.

To better understand this interaction, we additionally calculated post-hoc *t*-tests using a Bonferroni correction. As far as the important day is concerned, the changes back to internal processes happened more frequently after gaining new insights than after confirming previous knowledge *t*(40) = 4.57, *p* < 0.001, *d* = 1.08, 95% CI [0.25, 1.91], or after an unsuccessful consultation of an external resource, *t*(40) = 3.76, *p* = 0.002, *d* = 0.96, 95% CI [0.10, 1.82]. There was no significant difference for the number of changes after confirming previous knowledge and after an unsuccessful consultation, *t*(40) = 0.90, *p* = 1.00, *d* = 0.13, 95% CI [− 0.32, 0.57]. That is, for the important day, most changes back to internal processes happened after gaining new insights from an external resource, indicating that consulting an external resource was an overall successful strategy. As far as the random day is concerned, the changes back to internal processes happened more frequently after gaining new insights than after confirming previous knowledge, *t*(40) = 9.60, *p* < 0.001, *d* = 1.61, 95% CI [0.84, 2.38], or after an unsuccessful consultation of an external resource, *t*(40) = 2.57, *p* = 0.042, *d* = 0.62, 95% CI [− 0.16, 1.40]. Interestingly, the changes back to internal processes also happened more frequently after an unsuccessful consultation of an external resource than after confirming previous knowledge, *t*(40) = 4.76, *p* < 0.001, *d* = 1.00, 95% CI [0.26, 1.74]. That is, while consulting an external resource was also an effective strategy in the case of the random day, there was also a relatively high number of cases in which consulting an external resource was unsuccessful. In addition, the number of changes from an external resource to an internal process after gaining new insights did not differ significantly between the important day and the random day, *t*(40) = 1.31, *p* = 0.199, *d* = 0.28, 95% CI [− 0.39, 0.94]. However, there were more changes back to an internal process after an unsuccessful consultation of an external resource for the random day compared to the important day, *t*(40) = 3.12, *p* = 0.003, *d* = 0.62, 95% CI [0.04, 1.27]. There were also more changes back to an internal process after confirming previous knowledge for the important day compared to the random day, *t*(40) = 2.50, *p* = 0.017, *d* = 0.26, 95% CI [− 0.08, 0.59].

### Kinds of external resources

The descriptive results for the kinds of external resources that participants used are displayed in Tables 2 and 3. While Table 2 gives a detailed overview of the different kinds of external resources and the number of participants who used them, Table 3 shows the average number of digital, non-digital, and social resources that participants used in the two conditions. As the descriptive numbers clearly indicate, digital resources were used much more frequently than non-digital and social resources. Whereas the vast majority of participants used digital resources (38 of our 41 participants when remembering the important day and 40 participants when remembering the random day), non-digital (important day: 1 participant; random day: 2 participants) and social resources (important day: 1 participant; random day: 5 participants) were hardly used at all. Against this background, we decided to focus on the digital resources for this part of our statistical analysis. In this context, a *t*-test for dependent samples shows that participants used more digital resources when remembering the random day compared to remembering the important day, *t*(40) = 5.33, *p* < 0.001, *d* = 0.83, 95% CI [0.47, 1.19]. Interestingly, the digital resources that were used most frequently when remembering the important day (i.e., smartphone photo gallery, digital calendar, instant messaging, snapchat) were the same digital resources that were also used most frequently when remembering the random day, indicating that remembering a random day versus remembering an important day does not so much change the kind of resources that individuals use but rather the frequency with which these resources are consulted.

**Table 2:** Number of participants using the different external resources

	Important day	random day
*Digital resources*	38	40
Smartphone photo gallery	27	31
Digital calendar	18	30
Instant messaging	10	17
Snapchat	10	14
BeReal	3	3
E-mails	2	4
Internet search	2	6
Digital notes	2	4
University websites	2	4
Instagram	0	2
Local hard drive	0	6
Other	4	6
*Non-digital resources*	1	2
Printed calendar	0	1
Paper diary	1	1
*Social resources*	1	5
Family	0	2
Partner	1	3

The table displays the number of participants (*N* = 41) who used the different social, non-digital, and digital resources. The numbers next to the overarching categories (i.e., digital, non-digital, and social resources) indicate the number of participants who used *at least one* resource from this category

**Table 3:** Average number of different external resources used by participants

	Important day		Random day	
	M	SD	M	SD
Digital resources	2.02	1.47	3.22	1.92
Non-digital resources	0.02	0.16	0.05	0.22
Social resources	0.02	0.16	0.12	0.33

The table displays the average number of different external resources that participants (*N* = 41) used when remembering the important day and the random day

### Retrieved information

As already stated above, the amount of retrieved information was operationalized as the number of different pieces of information that participants retrieved for the important day and the random day based on internal memory and external resources, respectively. A detailed overview of the different types of information (i.e., the different kinds of internal states and external characteristics) for the different types of retrieval (i.e., internal versus external retrieval) and the different types of events (i.e., random day vs. important day) is reported in Table 4.

**Table 4:** Type of retrieval for different types of information across both types of events

	Internal retrieval				External retrieval			
	Important day		Random day		Important day		Random day	
	M	SD	M	SD	M	SD	M	SD
*External characteristics*								
Places	1.37	1.28	0.56	0.71	0.44	0.87	0.61	0.833
Events	5.05	2.56	2.37	2.40	0.93	1.13	1.49	1.23
Persons	2.76	1.85	0.95	1.53	0.63	0.94	0.63	0.86
Conversational content	0.56	1.23	0.46	1.00	0.66	1.70	0.51	1.10
*Internal states*								
Thoughts	0.22	0.65	0.24	0.66	0.00	0.00	0.02	0.16
Feelings	1.41	1.96	0.59	0.95	0.07	0.26	0.05	0.22
*Total*	11.37	7.05	5.17	5.39	2.73	3.62	3.32	2.93

We ran a 2 × 2  repeated-measures ANOVA to determine whether the *kind of event* (important day vs. random day) and the *kind of retrieval* (internal retrieval vs. external retrieval) influenced the *amount of retrieved information*. We found a significant main effect for the kind of event, *F*(1, 40) = 20.18, *p* < 0.001, $\omega_{p}^{2} = 0.{152}$, a significant main effect for the kind of retrieval, *F*(1, 40) = 29.68, *p* < 0.001, $\omega_{p}^{2} = 0.{295}$, and a significant interaction between the two factors, *F*(1, 40) = 22.64, *p* < 0.001, $\omega_{p}^{2} = 0.191$. As indicated by post-hoc *t*-tests using a Bonferroni adjustment, participants retrieved more information through internal retrieval for the important day compared to the random day, *t*(40) = 5.00, *p* < 0.001, *d* = 1.24, 95% CI [0.45, 2.02]; for external retrieval, however, there was no difference between the amount of information retrieved for the important day compared to the random day, *t*(40) = 1.15, *p* = 0.259, *d* = −0.12, 95% CI [− 0.40, 0.17]. In addition, participants retrieved more information through internal retrieval than through external retrieval when remembering an important day, *t*(40) = 6.60, *p* < 0.001, *d* = 1.72, 95% CI [0.82, 2.62]; when remembering a random day, however, there was no significant difference between the amount of information retrieved through internal retrieval and external retrieval, *t*(40) = 1.72, *p* = 0.092, *d* = 0.37, 95% CI [−0.24, 0.98].

### External resources and retrieved information

The higher the total number of different external resources (i.e., the higher the total number of social, non-digital, *and* digital resources combined), the more information they remembered through external retrieval, both when remembering the important day, *r* = 0.71, *p* < 0.001, 95% CI [0.51, 0.83], and when remembering the random day, *r* = 0.37, *p* = 0.018, 95% CI [0.07, 0.61]. The correlation for the important day is significantly stronger than the correlation for the random day, *z* = 2.17, *p* = 0.030. These results remain virtually unchanged when using the number of different digital resources (instead of the total number of different external resources): In other words, the more different digital resources participants used, the more information they remembered through external retrieval, both when remembering the important day, *r* = 0.72, *p* < 0.001, 95% CI [0.53, 0.84], and when remembering the random day, *r* = 0.39, *p* = 0.011, 95% CI [0.10, 0.63]. Again, the correlation for the important day was significantly stronger than the correlation for the random day, *z* = 2.13, *p* = 0.033. Similar to the analysis regarding the frequency of successful and unsuccessful consultations of an external resource, this analysis demonstrates that using external resources is effective, albeit less so for the random day.

## Discussion

The present study was designed to gain new insights into autobiographical remembering in the twenty-first century. In addition, it was also designed to test some of the key assumptions underlying our AMEDIA-Model (Hutmacher et al., 2024a, 2024b), namely the assumption that autobiographical remembering is an iterative process based on the combination of information stored in one’s mind and information stored in external resources and the assumption that digital external resources play an important role when it comes to remembering autobiographical events in the digital age.

### Summary of findings and theoretical implications

As far as the first assumption is concerned, our data convincingly demonstrate that individuals repeatedly change between internal memories and information stored in external resources, no matter whether they try to remember an important day or a random day. Three more observations are important in this context. First, our data do not only show that individuals rely on external resources for autobiographical remembering but also that this is an effective strategy: For both the important day and the random day, consulting an external resource more frequently led to new insights than it remained unsuccessful. In addition, the more external resources individuals used, the more information they retrieved from these external resources. Interestingly, this pattern was less pronounced for the random day compared to the important day as indicated by a higher frequency of unsuccessful consultations of an external resource as well as a weaker correlation between the number of external resources that participants used and the amount of information that they were able to retrieve. As we see it, there are at least two explanations for these findings. On the one hand, it could be that looking at different resources is less effective for the random day because the participants search strategy is less targeted. Assuming that participants have fewer internal memories about the random day compared to the important day, they may be less sure where to look for the respective information. On the other hand, it could be that the external resources simply contain fewer information for the random day compared to the important day so that consulting an external resource has a higher chance of remaining without a result.

Second, the amount of information that individuals were able to retrieve based on internal memories was higher when remembering an important day compared to remembering a random day. Note, however, that this pattern is difficult to interpret: It might be that individuals are able to retrieve more information for the important day *because* it was an important day, that is, because they ascribe a higher value to the things that happened during this day. At the same time, it is also possible that simply more happened during the important day so that the maximum amount of information than can be retrieved for the important day is higher than the maximum amount of information that can be retrieved for the random day. Interestingly, there was no significant difference between the important day and the random day regarding the amount of information that individuals were able to retrieve solely based on external resources. If anything, there was a descriptive tendency that more information was retrieved from external resources when remembering the random day. This suggests that external resources provide a valuable basis for remembering and reconstructing both important and comparably unimportant autobiographical events.

Third, we observed more changes between internal processes and external resources for the random day compared to the important day. In line with this, we also observed that individuals used more (digital) external resources and that they used these resources earlier when remembering the random day compared to remembering the important day. As remembering a random day based on internal memory alone is harder than remembering an important day, this seems to suggest that individuals tend to access external resources whenever the information stored in internal memory seems insufficient. This conclusion is also supported by our additional analyses demonstrating that individuals only rarely switch to an external resource when they already have a detailed internal memory, but rather when they have either no internal memory or merely a vague internal memory. Moreover, initiating a switch to an external resource because one had no internal memory was more frequent for the random day compared to the important day.

Taken together, these results indicate that the iterative combination of information stored in one’s mind and information stored in the environment is—at least potentially—complementary and symbiotic in the sense that it improves remembering compared to unaided and unmediated recall. This does not only support the foundations of the AMEDIA-Model (Hutmacher et al., 2024a, 2024b) but also of related frameworks (Eliseev & Marsh, 2021; Finley & Naaz, 2023; Finley et al., 2018), which all emphasize that external resources are not so much used for offloading and externalization but rather to create an extended memory system. In this context, it should be noted that retrieving information from external resources and retrieving information from internal memory are in fact not independent but constantly interact: On the one hand, the available internal memories may guide one’s search strategies when browsing external resources; on the other hand, the information found in external resources may make internal memories available that one would not have been able to retrieve without using external resources. In fact, and as described in the method section, internal retrieval—as understood in the present manuscript—comprised cases of unaided internal retrieval (e.g., “I remember being in a restaurant that day”) and aided internal retrieval (e.g., “Now that I see it on the picture, I also remember being in a restaurant that day”). Although we were not able to distinguish between these cases based on our think-aloud protocols, we encourage future research that is able to make these more fine-grained distinctions.

Apart from that, the data also confirm the assumption that digital resources play a key role for autobiographical remembering in the digital age. More specifically, the number of digital resources that individuals used was much higher than the number of non-digital and social resources. To a certain degree, this could of course be a result of the study setting (i.e., a videoconferencing tool being used for recording both sessions). At the same time, participants were explicitly told that they are allowed to stand up, leave the room, and talk to others to minimize such effects. That is, although we caution against overinterpreting the relative frequency with which participants made use of social, non-digital, and digital tools, the importance of digital tools for autobiographical remembering in the twenty-first century seems undeniable. As it has been shown that remembering and narrating autobiographical events often takes place in conversation with others (Grysman et al., 2024; Nelson & Fivush, 2004; Pasupathi & Wainryb, 2010), however, it could nevertheless be enlightening to repeat the study in different settings. With respect to digital resources, one more interesting finding emerged: The most frequently used digital resources were the same for remembering the important day and the random day. This implies that individuals have their preferred sources for storing and retrieving information about their lives that they consistently use under varying circumstances.

### Limitations and constraints on generality

We note three limitations. First, our results could be influenced by the choice of the days that we compared. We picked the participants’ (second) last birthday as the important day to be remembered as we assumed that most participants would find their birthday important and consider it a day that stands out from the rest of the year. Nevertheless, future research could explore different kinds of important events to test the generalizability of our results. Also note that the important day was the same for all participants (namely their last birthday), while the random day could be anything so that the comparison between the important day and the random day ultimately is a comparison of one specific important day with many different random days.

Second, the results could also be influenced by the specifics of our instructions. Our main goal was to create a study setting that is much closer to real-world scenarios than many other studies on autobiographical remembering in that it did not isolate individuals from their environment and but invited them to use the available external resources. In line with this, we gave our participants a maximum of 15 min to reconstruct each day to restrict the session to a reasonable length, but also emphasized that participants can finish the task earlier if they have the impression that there is nothing more to be remembered. In addition, participants were told that they are allowed to use external resources; whether and to what degree they decided to do so, however, remained completely up to them. That being said, the participants’ task was to reconstruct the two days *in as much detail as possible*. Although this is something that participants may decide to do in real-world situations, both for an important day (e.g., “How exactly did I celebrate my last birthday?”) and for a random day (e.g., “What did I do Friday afternoon two weeks ago?”), one can easily imagine different instructions. For instance, participants could be instructed to reflect upon or reminisce about past events (cf. Sellen & Whittaker, 2010) rather than to reconstruct them in as much detail as possible. Alternatively, one could also ask participants to remember events that are not related to a specific pre-defined date as in the present study (e.g., the day one’s first child was born, the last time one had pizza with friends) and that might therefore require a different way of accessing and using external resources.

Third, our study addressed the interplay between internal *autobiographical* memory and external resources. As outlined in previous research, however, individuals do not only make use of external resources for autobiographical memory purposes, but also for semantic, procedural, and prospective purposes (Finley & Naaz, 2023). Importantly, the frequency and ease of use varies across these different memory purposes, suggesting that the degree to which individuals are willing and able to use external resources may depend on the goal that they have in mind. Hence, we caution against overinterpreting our results: More research is needed to determine whether the same patterns can also be observed for non-autobiographical memory purposes.

### Directions for future research

Although the present study provided empirical evidence for some of the key claims underlying the AMEDIA-Model (Hutmacher et al., 2024a, 2024b), many open research questions remain. In addition to the limitations already discussed above, we want to mention two points: First, the AMEDIA-Model hypothesizes that the way data have been encoded and curated influences how the respective autobiographical events are remembered later (see also Henkel & St. Jacques, 2024). In the present study, participants combined all available external resources to reconstruct the two days in as much detail as possible. Nevertheless, it would be interesting to know which separable effects different resources have on remembering past events. In addition, it could be insightful to compare conditions in which individuals have access to (different) external resources to a control condition, in which they do not, in order to capture the full potential of having a digital database of our lives. This might also include checking the reliability of different sources and the probability with which they might lead to false recognitions and distorted reconstructions (cf. St. Jacques & Schacter, 2013). Second, we asked participants to remember events that they experienced about one year ago. One potential avenue for future research could be to extend this time interval. This could be particularly interesting as file formats and the applications that individuals use have undergone constant changes over the last years and decades (e.g., Blum & Beyer, 2019; see also Petrelli & Whittaker, 2010; van den Hoven et al., 2012; van House & Churchill, 2008), suggesting that it could be more difficult to reconstruct autobiographical events using external resources from the more distant past. In a similar vein, it could also be interesting to shorten the time interval. Ultimately, using different time intervals would enable comparisons between the ways internal memories and external resources are used to reconstruct autobiographical events from one month, one year, or one decade ago.

### Conclusion

In sum, the present study provides compelling evidence for the importance of considering the interplay between information stored in our minds and information stored in external resources as well as the role of digital resources when it comes to autobiographical remembering in the twenty-first century. Although humans have used external resources for remembering for thousands of years already, the proliferation of digital technologies has significantly transformed the memory ecologies in which humans are embedded. Further exploring how this shapes the way we remember our lives will be an important task in the coming years.

## Supplementary Information

Supplementary Material 1

## Data Availability

Quantitative data, study materials, and the codebook that was used for analyzing the qualitative data are available at https://osf.io/n2t7u/.

## Footnotes

1 We are indebted to the editor and the reviewers for suggesting these more fine-grained analyses during the revision process.

2 Naturally, this *t*-test only involved participants who used at least one external resource per session. This applied to 37 of the 41 participants.

3 We decided to report omega squared instead of the more common eta squared because omega squared can be considered to be less biased (for details, see Kroes & Finley, 2023).
