# Supplementary material for: Mediated autobiographical remembering in the digital age: insights from an experimental think-aloud study
Source: Cogn Res Princ Implic. 2025 May 1;10:18. doi: 10.1186/s41235-025-00627-4 (PMC12045852; doi:10.1186/s41235-025-00627-4)
Supplement: Supplementary file 1 — Supplementary Material 1 [file 41235_2025_627_MOESM1_ESM.docx]

**Online Supplement for**

**“Mediated autobiographical remembering in the digital age: Insights from an experimental think-aloud study”**

As also reported in the manuscript, the time that participants spent on performing the think-aloud procedure did not differ significantly between remembering the important day (*M* = 6.87 minutes, *SD* = 3.11) and remembering the random day (*M* = 7.47 minutes, *SD* = 3.45), *t*(40) = 1.31, *p* = .098, *d* = 0.21, 95% CI [-0.11, 0.51]. As there was a descriptive tendency that participants spent more time on remembering the random day, we nevertheless checked whether correcting the parameters of interest for the time spent on the task changes the results in any meaningful way. This was not the case: All significances remained the same. In the following, we report these additional analyses for the adjusted values.

**Changes between Internal Memory and External Resources**

We calculated the number of changes per minute spent on the task for each participant. Overall, and as indicated by a t-test for dependent samples, participants changed more frequently per minute between internal memory and external resources when remembering the random day (*M* = 1.10, *SD* = 0.55) compared to remembering the important day (*M* = 0.83, *SD* = 0.45), *t*(40) = 3.13, *p* = .003, *d* = 0.49, 95% CI [0.16, 0.81].

**Kinds of External Resources**

We calculated the number of different digital resources that participants used per minute spent on the task. In this context, a t-test for dependent samples shows that participants used more digital resources per minute when remembering the random day (*M* = 0.46, *SD* = 0.23) compared to remembering the important day (*M* = 0.30, *SD* = 0.18), *t*(40) = 4.35, *p* < .001, *d* = 0.68, 95% CI [0.34, 1.02].

**Retrieved Information**

We calculated the average amount of information retrieved per minute based on internal retrieval versus external retrieval for both kinds of events (important day vs. random day). Next, we ran a 2×2 repeated-measures ANOVA to determine whether the kind of event (important day vs. random day) and the kind of retrieval (internal retrieval vs. external retrieval) influenced the amount of retrieved information. We found a significant main effect for the kind of event, *F*(1, 40) = 47.26, *p* < .001, *η_p_²* = .54, a significant main effect for the kind of retrieval, *F*(1, 40) = 34.94, *p* < .001, *η_p_²* = .46, and a significant interaction between the kind of event and the kind of retrieval, *F*(1, 40) = 31.28, *p* < .001, *η_p_²* = .44. As indicated by pairwise comparisons using a Bonferroni adjustment, participants retrieved more information per minute through internal retrieval for the important day (*M* = 1.86, *SD* = 1.12) compared to the random day (*M* = 0.80, *SD* = 0.86), *p* < .001, *d* = 2.24; for external retrieval, however, there was no difference between the amount of information retrieved per minute for the important day (*M* = 0.35, *SD* = 0.41) compared to the random day (*M* = 0.50, *SD* = 0.55), *p* = .148, *d* = 0.47. In addition, participants retrieved more information per minute through internal retrieval than through external retrieval when remembering an important day, *p* < .001, *d* = 2.29; when remembering a random day, however, there was no significant difference between the amount of information retrieved per minute through internal retrieval and external retrieval, *p* = .073, *d* = 0.58.
